# Thymic Hassall’s corpuscles in Nandanam chicken - light and electronmicroscopic perspective (*Gallus domesticus*)

**DOI:** 10.1186/s40781-015-0064-2

**Published:** 2015-10-01

**Authors:** Thandavan Arthanari Kannan, Geetha Ramesh, S. Ushakumary, Gopalan Dhinakarraj, Subbiah Vairamuthu

**Affiliations:** Centre for Stem Cell Research and Regenerative Medicine Madras Veterinary College, Tamil Nadu veterinary and Animal Sciences University, Chennai, 600 007 India; Department of Veterinary Anatomy, Madras Veterinary College, Chennai, 600 007 India; Translational Research Platform and Veterinary Biological Tamil nadu Veterinary and Animal Sciences University, Chennai, India; Central Clinical Laboratory Madras Veterinary College, Chennai, 600 007 India

**Keywords:** Light and electronmicroscopy, Thymus, Hassall’s corpuscles, Nandanam Chicken

## Abstract

The present study was aimed to study the light and electron microscopic studies of thymic Hassall’s corpuscles was done in various age groups of Nandanam Chicken ranging from day-old to forty weeks. Hassall’s corpuscles are special, unique structures present in thymic medulla and also in the cortex of all the age groups of Nandanam chicken (from hatch to forty weeks) in the present study. Size of the Hassall’s corpuscles in the medulla is larger than the ones present in the cortical region of thymus. The Hassall’s corpuscles are made up of structureless eosinophilic mass surrounded by concentrically arranged reticuloepithelial cells. Under electron microscope, the Hassall’s corpuscles were composed of reticuloepithelial cells interconnected by many desmosomes. The epithelial cells had abundance of cytoplasmic fibrils and desmosomes with few mitochondria and ribosomes. The nucleus was oval or round which was slightly indented. The centre of the Hassall’s corpuscles was appeared either solid or cystic. The cystic corpuscles had cell debris within the cyst lumen.

## Background

All living beings manage not only to survive but indeed thrive in potentially hostile milieu, without seeming effort. This freedom from disease is depends on the existence of a complex and highly sophisticated defense system, called lymphatic system [1, 2].

Thymus is a central lymphoid organ in which bone marrow-derived T-cell precursors undergo differentiation, maturation and eventually leading to migration of positively selected thymocytes to the peripheral lymphoid organs [3, 4]. Thymus differs from other lymphoid organs as it undergoes involution as age advances [5].

In chicken, thymic parenchyma is madeup of an outer darker cortex and inner paler medulla [6]. Hassall’s corpuscles are characteristic structure of thymic medulla, first demonstrated by Arthur Hill Hassall who described it as an acidophilic, squamous spherical structures in the thymic medulla, unique to this organ [5, 7–9].

There are several studies highlighted the existence of differences between thymic Hassall’s corpuscles of mammals and birds [10–12]. Nandanam chicken is a dual purpose, colored variety with good disease resistance and most popular among poultry farmers due to its adaptability to backyard farming. This strain was developed in Institute of Poultry Production and Management, Tamil Nadu Veterinary and Animal Sciences University, Chennai. Hence, the present study has been designed with aim of exploring the light and electronmicroscopic structural details of thymic Hassall’s corpuscles in Nandanam chicken.

## Methods

Over 36 specimens of thymic tissue were collected from six different age groups such as day-old, four, eight, twelve, twenty and forty weeks. Six birds were used in each age group. The thymus was removed immediately after high cervical dislocation and fixed for light and electron microscopy [13].

For light microscopic studies, tissue pieces were fixed in 10 % neutral buffered formalin and processed for paraffin (Cat. No. 8002-74-2 Sigma-Aldrich, India) embedding technique [14]. Tissue sections were cut at 5 micron thickness and used for routine Haematoxylin-eosin staining method [15].

For electronmicroscopic study, small pieces of thymic tissue (1–2 mm thickness) were collected and prefixed at 3 % glutaraldehyde (Cat. No. G-5882, Sigma-Aldrich, India) and stored at 4 °C. Subsequent processing, sectioning of tissue and staining was done as per [16]. The ultra thin sections were examined under Phillips (Teknai-10) computer augmented transmission electron microscope operated at 60-kilowatt ampere (KVA).

Procurement of birds for this study was approved by Institutional Animal Ethical Committee, Madras Veterianry College, Chennai-07.

## Results and discussion

### Light microscopy

**Fig. 1:**
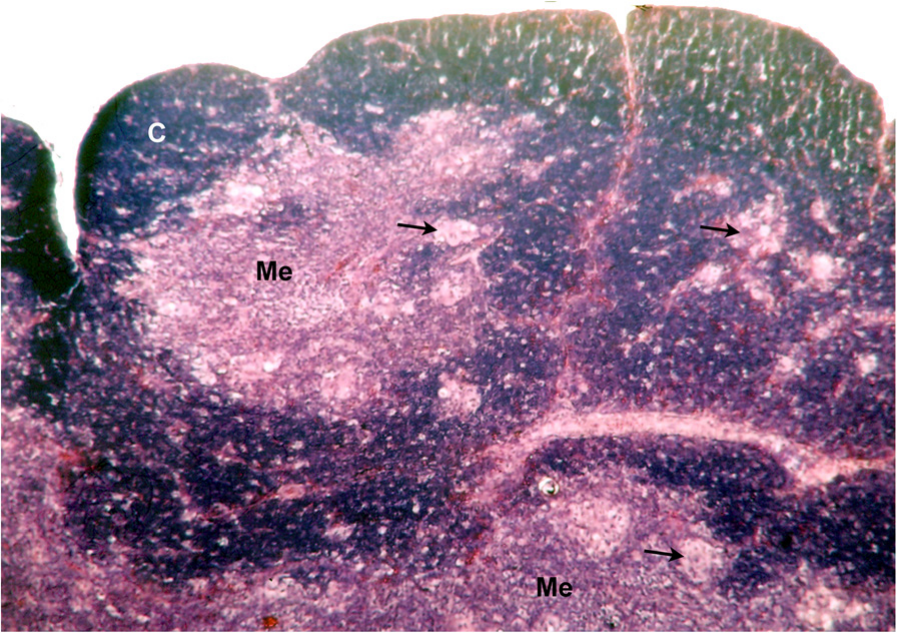
Photomicrograph of thymus of four week-old chicken showing cortex and medulla with Hassall’s corpuscles (arrows) H&E x 100. C - Cortex, Me - Medulla

In the present study, the Hassall’s corpuscles were commonly were found to be round, homogenous eosinophilic mass lined by flat reticuloepithelial cells observed in the medulla of chicken thymus in all age groups studied [17–19, 12]. However, the presence of Hassall’s corpuscles was also noticed in the cortical areas of day-old, four weeks and ten weeks age groups (Fig. 1). The corpuscles present in the cortex were smaller in size with few cells whereas the medullary ones were larger, madeup of hyalinized eosinophilic mass at the centre and peripheral concentrically arranged reticuloepithelial cells [18, 20].

The number of corpuscles was found to be more as age advanced in the present study [19] which indicated the involutory changes in aged birds [21]. The corpuscles were normally associated with blood sinusoids.

**Fig. 2:**
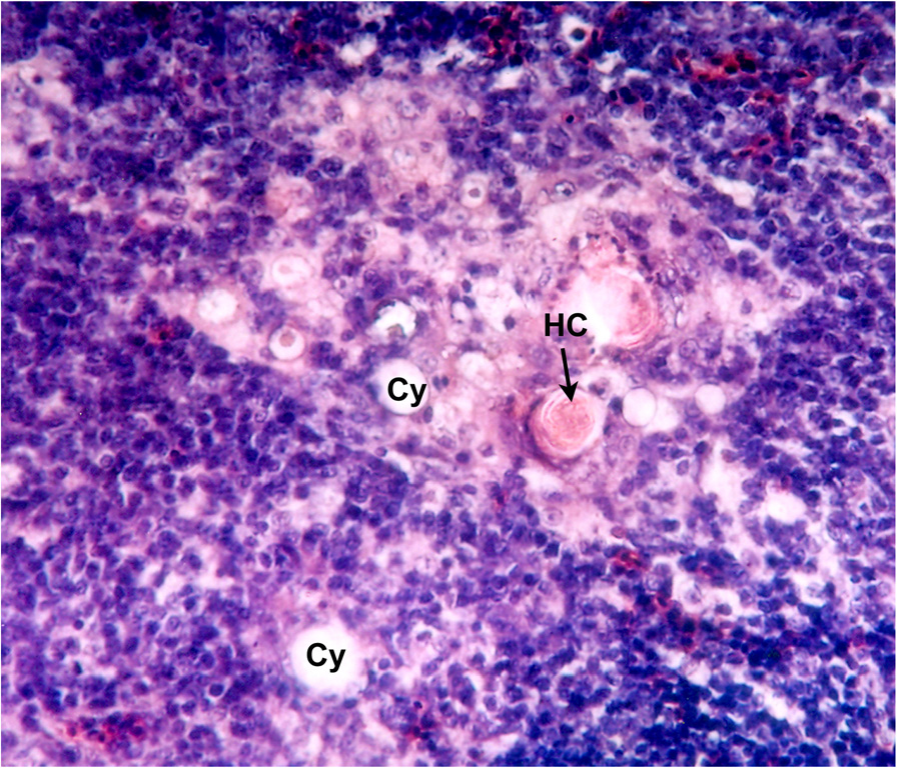
Photomicrograph of the thymic parenchyma of a day-old chick H&E x 400. Cy - Cyst, HC - Hassall’s corpuscle

The presence of unicellular cysts were common in all the age groups studied which occurred either free in the parenchyma or associated with the Hassall’s corpuscles [22, 23]. Multicellular cysts were also noticed in the advanced age groups of chicken in the present study (Fig. 2).

### Electronmicroscopic study

**Fig. 3:**
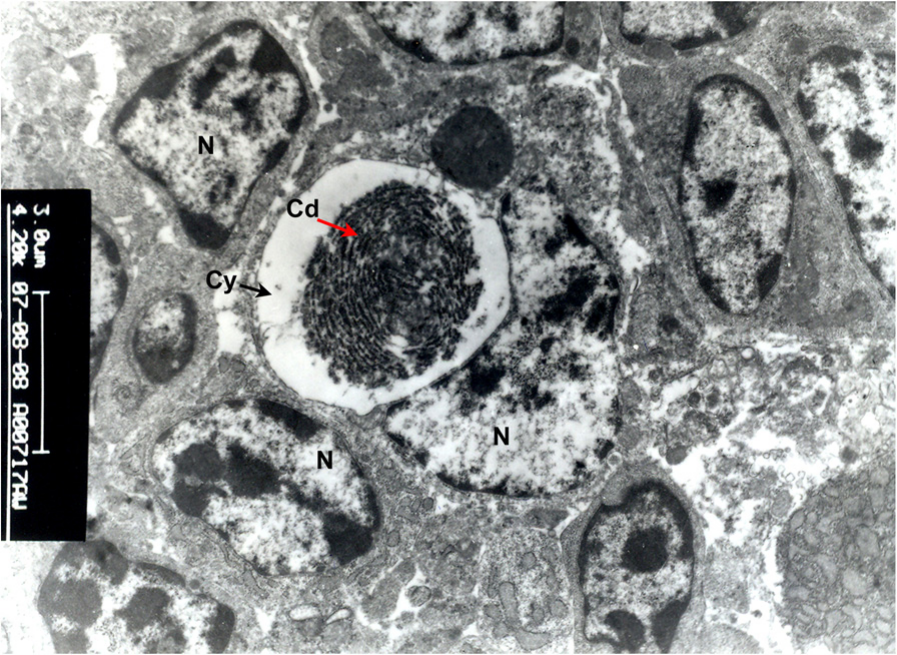
Transmission electronmicrograph of thymus of an eight week-old chicken showing Hassall’s corpuscles in the medulla x 4200. Cd - Cell debris, Cy - Cyst, N - Nucleus of reticuloepithelial cell

**Fig. 4:**
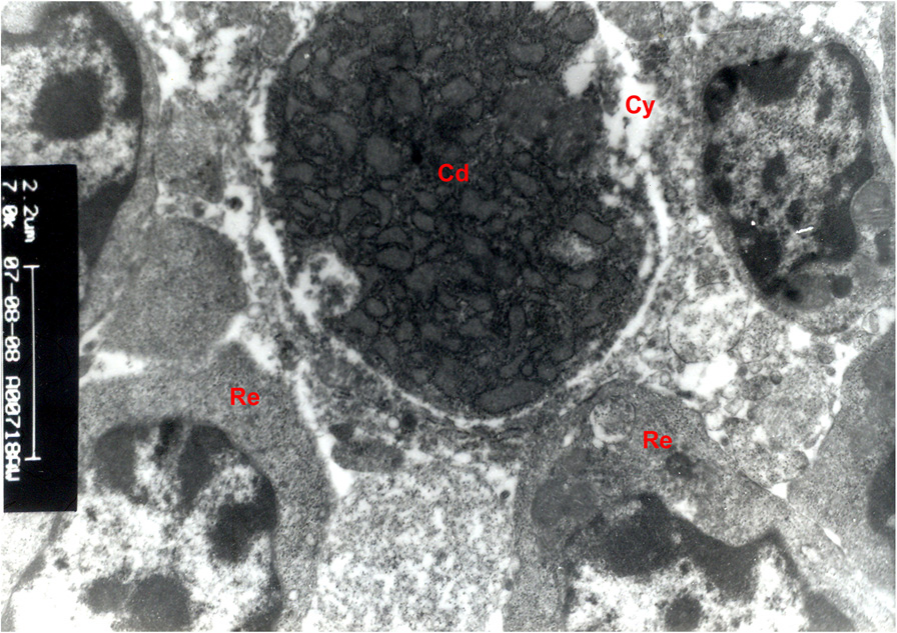
Transmission electronmicrograph of thymus of an eight week-old chicken showing the Hassall’s corpuscles x 7000. Cd - Cell debris, Cy - Cyst, Re - Reticuloepithelial cell

Under electron microscope, the Hassall’s corpuscles were composed of reticuloepithelial cells interconnected by many desmosomes. These epithelial cells had abundance of cytoplasmic fibrils and desmosomes with few mitochondria and ribosomes. The nucleus was oval or round which was slightly indented (Fig. 3). A nucleolus was also observed. The centre of the Hassall’s corpuscles was appeared either solid or cystic. The cystic corpuscles had cell debris within the cyst lumen (Fig. 4). However, some of them were seen empty. The lining epithelial cells had microvilli [24]. In contrast, the large Hassall’s corpuscles were not often seen in the chick thymus, when present they were composed of concentric rings of squamous epithelial cells interconnected by many desmosomes [25].

The association of dieing cells and macrophages with Hassall’s corpuscles in the present study proved beyond doubt that Hassall’s corpuscles were the repository for a great number of old cells [26, 27]. This finding is contrary to the findings in guinea pig where they opined that the Hassall’s corpuscles are the privileged areas for maturation of the medullary lymphocytes [28].

In the present study, two types of vesicles or cysts were observed in association with the Hassall’s corpuscles in all the age groups. Many epithelial cells in the medulla formed the unicellular or intracellular and multicellular or intercellular cysts. The cytoplasm of the intracellular cysts contained mitochondria, endoplasmic reticulum, Golgi apparatus, ribosomes, some small dense granules. The nucleus was pale, oval and had one or two nucleoli. These cystic cells were found attached to the neighbouring epithelial cells by desmosomes.

The intercellular cysts were found to be formed by two or three cells which varied from very pale to very dark in their appearance. The cytoplasm contained a few mitochondria and ribosomes and very little rough endoplasmic reticulum. The nucleus was found to be oval or spherical. The microvilli was seen projected into the lumen of the cyst. Some of the cysts appeared to be empty while some of them contained mucous droplets in this type of cyst also [29].

In the present study, the cells of the intercellular cysts contained dense secretory granules which are similar to the findings in chicken [25]. This is good evidence to support the idea that the thymus is an endocrine gland, and secretes a hormone that induces lymphoid cells to acquire immunological competence [30]. Whereas, it was opined that that cystic epithelial cells showed cytoplasmic features suggestive of a secretory function [31, 32].

Also some evidence to suggest that cystic epithelial cells in the mouse thymus secrete a sulphated mucoid lymphopoietic hormone [33]. It was reported that a mucoid substance is regularly secreted by the epithelial cells forming the Hassall’s corpuscles in the human thymus [34]. Whereas, it was suggested that “clear vesicles” present in reticulum cells of the rat thymus are the morphological equivalent of the production of a humoral thymus factor [35]. However, [36, 37] considered that the granular inclusions in cystic epithelial cells may be related to a lymphocyte stimulating hormone. It was suggested that dense granules present in epithelial cells in the guinea pig thymus were kerato-hyaline granules linked to keratinisation but he did not exclude the possibility of the presence of another form of endocrine secretion, especially a colloid or steroid secretion [38].

## Conclusions

Hassall’s corpuscles were unique structure of thymus, present in both cortex and medulla. Number of Hassall’s corpuscles increased as age advances. The corpuscles were madeup of homogenous, eosinophilic mass surrounded by concentrically arranged reticuloepithelial cells. In the present study, two types of cysts, intracellular and intercellular cysts were observed in association with the Hassall’s corpuscles in all the age groups. Intracellular cyst was either free or associated with the Hassall’s corpuscles were common in all the age groups. Intercellular cysts were noticed in advanced age groups of chicken.
